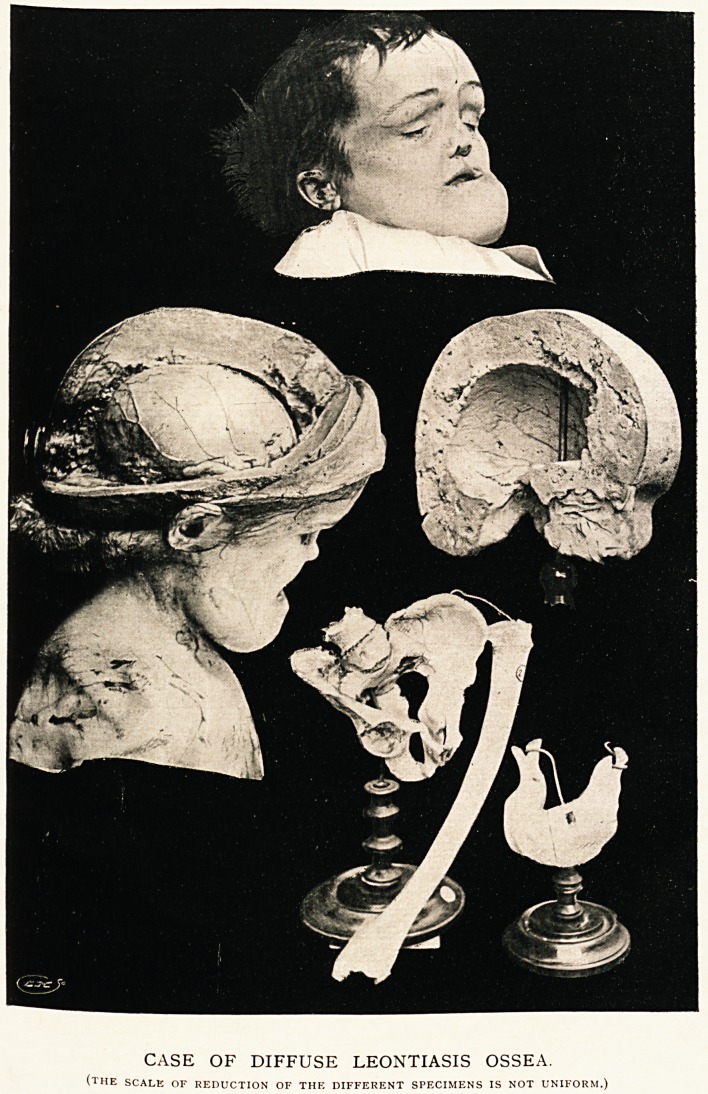# A Case of Diffuse Leontiasis Ossea

**Published:** 1900-12

**Authors:** E. H. Edwards Stack

**Affiliations:** House Physician, Royal Infirmary, Bristol


### A CASE OF DIFFUSE LEONTIASIS OSSEA.

BY

E. H. EDWARDS STACK, M.B., F.R.C.S.,

*House Physician, Royal Infirmary, Bristol.*

THIS very uncommon case is probably one of leontiasis ossea of the diffuse variety. Ziegler[1] calls it "partial gigantism," which "affects the bones of the cranial vault as well as those of the face. The overgrowth is sometimes uniform, at other times irregular. . . . Virchow has termed the condition leontiasis ossea."

The ætiology of this disease is obscure. The enlargement of the bones must be either simple hypertrophy, new growth, or chronic osteitis. The appearance negatives either of the first two, and taking the last view, there is some difference of opinion as to the cause. The majority of cases recorded have had a history of injury, but without being sufficiently definite to be put forward as a certain cause; in some cases the malady seems to be quite clearly connected with the injury.

[1] *Special Pathological Anatomy*, Sect. i.—viii., 1896, p. 208.

CASE OF DIFFUSE LEONTIASIS OSSEA.

(THE SCALE OF REDUCTION OF THE DIFFERENT SPECIMENS IS NOT UNIFORM.)

Bland Sutton thinks that rickets is the fundamental cause, and in this case the scoliosis and pelvis might be regarded as support in this direction, but the appearances were not those of rickets. They were both probably static in origin and due to the weight of the head. There is no tendency in the pelvis or in any of the long bones of the lower limbs towards buttress formation, which is so common in rickets.

In this case there was an injury when the child was only a few years old, which took some months to heal; but whether this could have been the starting-point of a very slow and spreading inflammatory process or not, it is impossible to say.

F. K., female, aged 21, born in 1878. Admitted to Royal Infirmary, Bristol, in December, 1899, for sore throat and dyspnœa, from which she died suddenly the same evening, tracheotomy failing to restore breathing.

Father and mother alive and healthy. They were not blood-relations. Mother has had eight children, this one being the second; labour a little tedious, otherwise natural. She has had three miscarriages since. No evidence of syphilis in parents or other children. No other member of the family on either side similarly affected. No phthisis or mania.

As a baby there was nothing abnormal noticed. When three years old she fell, cutting her forehead, for which she was in the General Hospital for several weeks, and afterwards as an out-patient for about a month; the wound, which had been suppurating, had then healed, and gave no further trouble. When seven years old, the mother first noticed the child's head was larger than natural; but this must have been developing for some time, as next year the child was taken from school on account of remarks made by the other children, and as it has always grown larger so slowly, it could not have developed enough for these remarks in one year. As a child she was mentally as other children, and has never been in this respect deficient. When twelve years of age, she was in the Royal Infirmary, for dacryo-cystitis, under Mr. Richardson Cross, and at this time was supposed to have hydrocephalus. The canaliculus of the right eye was slit up, "but a probe could not be passed on account of abnormality of nasal bones." When 19 she was admitted again, under Mr. Paul Bush, having fractured, by a slight fall, the lower end of the right femur, and at the same time dislocated the right patella outwards. There was synovitis of the knee-joint. A month after admission there was no union, but a month later she went home with the fracture firmly united. Since then she has occasionally been to the Infirmary for some slight ailment, and the head has been noticed to have gradually grown larger. The first recorded measurements were in 1897, when the bi-temporal diameter was $8\frac{3}{4}$ in., and the horizontal circumference from this spot was $30\frac{3}{4}$ in. From the most prominent part of occipital bone to glabella was $21\frac{1}{2}$ in., and the diameter between these two points $10\frac{3}{4}$ in. In 1899 the only recorded measurement which corresponds was the circumference in the same region, and it was then $31\frac{1}{4}$ in. The lower-jaw deformity, the mother considers, began about the same time as the head. For the last six or seven years she had always stayed at home, and was a great help to her mother in looking after the children, but never was any use in housework, as she tired easily, and when sitting down often rested her head against the wall or laid it on the table. She never had any fits. In December, 1899, she came for the last time to the Royal Infirmary. For some days before admission she had a sore throat and difficulty in swallowing. She was admitted under Dr. Waldo. Her condition then was very serious; she had a great deal of inspiratory dyspnœa. The palate was pushed forward by something firm and was inflamed. At first sight the girl seemed to have enormous hydrocephalus, but the eyeballs were not displaced downwards or forwards, and the head felt much harder and heavier than it does in that disease. The left side of the lower jaw was much enlarged from the symphysis to the angle, and the right side was also enlarged along the horizontal ramus. She had a double lateral curvature and rotation of the vertebræ, with corresponding deformity of the chest. The femora and tibiæ were bent somewhat, and there was flat-foot on both sides. The knee-jerks could not be obtained. The terminal joints of all the fingers, especially the third and fourth, were deflected towards ulnar side, and were also partially luxated dorsally and had very deficient movement. The thumbs were not affected. Sight, hearing, and smell were natural.

*Post-mortem Examination.*—The skull was enormously and almost uniformly thickened. All the bones were affected, including those of the face. The cerebellar portion of the skull was very protuberant, dipping down towards the neck, and measured in thickness here almost three inches. The forehead was very large and rounded. The orbits very deep from before backwards, but diminished in size vertically and horizontally. The foramina in the skull did not appear anywhere to be reduced in size. The bones of the skull on section were for the most part softer than natural, but the appearance was not quite homogeneous. In parts there were masses of more compact tissue, and in others the rarefaction had proceeded to such an extent as to leave spaces of an eighth of an inch in diameter. The half skull without the lower jaw weighed seven pounds and a half. The lower jaw weighed one pound. The least affected portion was the occipital bone just round the foramen magnum. The interior of the skull looked fairly natural, except that the pituitary fossa was partially obliterated.

The lower jaw had a few temporary teeth on the upper surface in the region which previously had been the alveolar ridge, and several of the permanent teeth were lying buried in the under-surface of the bone, quite on its lower aspect. The whole of the horizontal ramus was enlarged and rounded; the compact shell was expanded and filled with very porous bone, containing a quantity of very slimy, gummy material, which poured out when cut into. The upper parts of the ascending ramus and coronoid process were nearly natural.

The vertebræ presented the usual changes of scoliosis, but otherwise they and the ribs had a normal consistency. The pelvis was very light, very soft, and much distorted; it was markedly generally contracted, scoliotic, funnel-shaped and beaked. The femora were both bent and a little softer than natural. The tibiæ were also bent, and in some places the external compact layer was replaced by softened bone developed from the periosteum. Both feet had their arches flattened. There was no overgrowth in any bone apart from the head. The sternum and ribs seemed natural on section and did not break more easily than usual. The naso-pharynx contained a firm, white, new growth, attached to the hard and soft palate on their posterior surface, and extending into the right parotid region. The brain weighed 47 ozs. and was natural. Pituitary body normal. The thyroid had a mass resembling new growth in the right lobe.

A section of the naso-pharyngeal growth shows it to be a fibro-sarcoma, containing numerous myeloid cells. This must be regarded as something quite apart from the bone condition. The mass in the right lobe of the thyroid gland on section somewhat resembles the thyroid in exophthalmic goitre, in that the alveoli are lined with several layers of cubical cells, projecting in some places nearly a third of the way into the alveolus.

The organs in the chest and abdomen showed nothing noteworthy, except the liver. The pelvic organs were natural. The liver contained several round nodules, the size of small marbles, which looked like new growth, otherwise its appearance and weight were natural and fat was not suspected. The liver shows very extensive fatty infiltration and diffuse cirrhosis, the new small-celled infiltration surrounding small masses of detached liver-cells ranging from five or six to twenty or so. The portion which was thought, naked eye, to be a new growth has a fibrous sheath, but otherwise presents the same appearance as the rest of the section.

The possibility of the osteitis being syphilitic must be considered, and is impossible to negative. Sections of the liver resemble congenital syphilis more than anything else.

The other diseases which one must consider are multiple myelomata or myelopathic albumosuria. The urine was not examined for albumoses, as she died the evening of admission; but although there was softening of the bone, with fragility, the growth of the skull and the age of this patient are quite unlike such cases. They have been described as "general lymphomatosis of bones, occurring in old men, with increasing kyphosis, severe pains in the bones, and progressive debility."

Paget's disease, which the thickening of the skull at first suggested, does not occur in children, does not affect the bones of the face, and generally is well marked in some of the long bones.

Acromegaly affects hands and feet as well as face-bones and not so much the cranium, and the pituitary body was not enlarged. There is almost nothing suggestive of cretinism or of achondroplasia in this case.

The author has to thank Dr. Waldo and Mr. Paul Bush, under whose care this case was, for their kind consent to his publishing it; also Mr. J. C. Clayton for the excellent photographs taken of it; and Dr. S. V. Stock, who kindly prepared the microscopic sections.

## Figures and Tables

**Figure f1:**